# Soil erosion and lateral carbon fluxes from corn stover-derived biofuel

**DOI:** 10.1038/s41598-025-99218-y

**Published:** 2025-05-26

**Authors:** Xuesong Zhang, Stephen D. LeDuc, Seungdo Kim, Bruce E. Dale, Kaiguang Zhao, Yuyu Zhou, Gregory W. McCarty, Glenn E. Moglen

**Affiliations:** 1 USDA-ARS Hydrology and Remote Sensing Laboratory, Beltsville, MD 20705-2350 USA; 2 Office of Research and Development, U.S. Environmental Protection Agency, 109 TW Alexander Dr., Research Triangle Park, NC 27711 USA; 3 Great Lakes Bioenergy Research Center, Michigan State University, East Lansing, MI 48824 USA; 4 Chemical Engineering and Materials Science, Michigan State University, 3815 Technology Boulevard, Lansing, MI 48910 USA; 5 Environmental Science Graduate Program, School of Environment and Natural Resources, The Ohio State University, Columbus, OH 43210 USA; 6 Department of Geography and Institute for Climate and Carbon Neutrality, The University of Hong Kong, Hong Kong, China; 7 Department of Civil and Environmental Engineering, The University of North Carolina at Charlotte, Charlotte, NC 28223 USA

**Keywords:** Carbon cycle, Environmental impact

## Abstract

Crop residues hold promise to alleviate food vs. fuel competition and contribute to biofuel production. However, the impacts of lateral sediment and carbon fluxes caused by residue removal are not fully understood. Here we employ agroecosystem modeling to conservatively estimate lateral sediment and carbon fluxes resulting from partial corn stover removal in the U.S. Midwest. Results show substantial increases in soil erosion resulting from corn stover removal. For example, the area of continuous corn and corn soybean cropping systems exceeding soil erosion tolerance threshold could increase from 1.1 to 13.3% because of 66% corn stover removal. Depending on removal intensity, conservation, and crop rotation, the stover removal-induced increases in eroded soil organic carbon is equivalent to 3.9–12.5 gCO_2_e MJ^−1^, which is comparable to other components of the life cycle impacts of corn stover-derived biofuel. Our findings highlight the need to consider the soil erosion and lateral carbon fluxes impacts of corn stover removal in designing supply chains for cellulosic biofuel production.

## Introduction

**Fig. 1:**
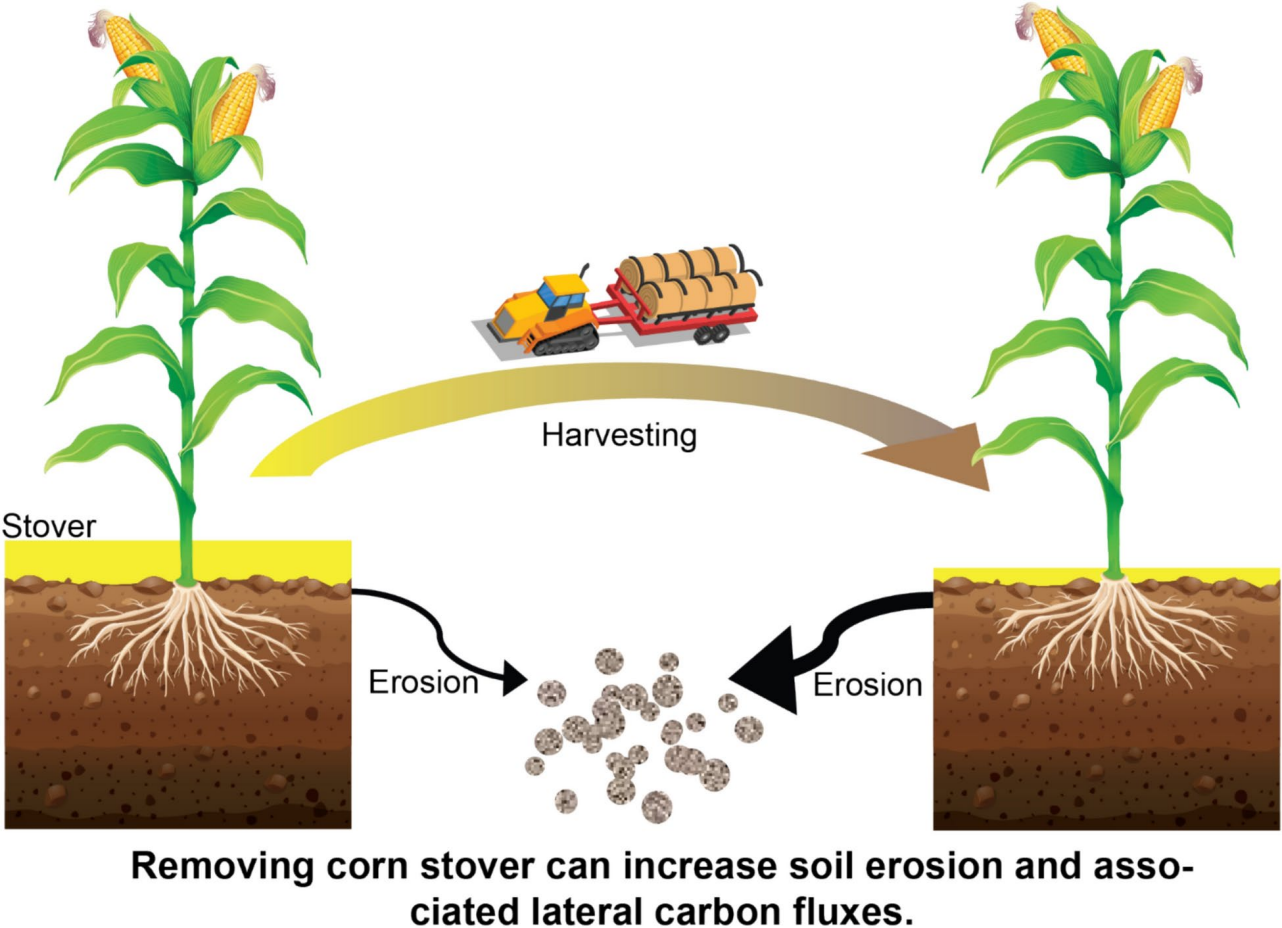
Schematic showing the effects of stover removal on soil erosion.

Bioenergy is a promising measure to support sustainable agricultural and energy development,^1,2^ but it can have unintended and unforeseen environmental consequences. In the United States, over 40% of corn (*Zea mays* L.) grain production has been directed to bioenergy production,^3^ raising serious concerns over food vs. fuel competition, water quality degradation, biodiversity loss, and indirect land use change.^4,5^ There is an urgent need for improved bioenergy production for environmental benefits. Crop residues are recognized as an important biofuel feedstock contributing to sustainable food and energy production.^6–8^ Corn stover is a readily available cellulosic bioethanol feedstock that holds promise to contribute to bioenergy production without directly competing with food supply.^9^ A range of 27 to 111 million tons of corn stover could be available for annual harvest in the United States, with most of the feedstock supply coming from the US Midwest or Corn Belt.^10,11^ However, collecting corn stover in erosion-susceptible locations could substantially increase soil erosion,^12^ which is a widespread threat to ecosystem sustainability across the globe.^13,14^ In addition, water erosion causes large lateral fluxes of carbon globally, up to 6.0 PgC yr^−1^ (or 22 PgCO_2_ yr^−1^).^15^ The economic cost and lateral carbon fluxes induced by soil erosion are not well understood for sustainable development of corn stover-derived cellulosic biofuel (Fig. 1).

**Fig. 2:**
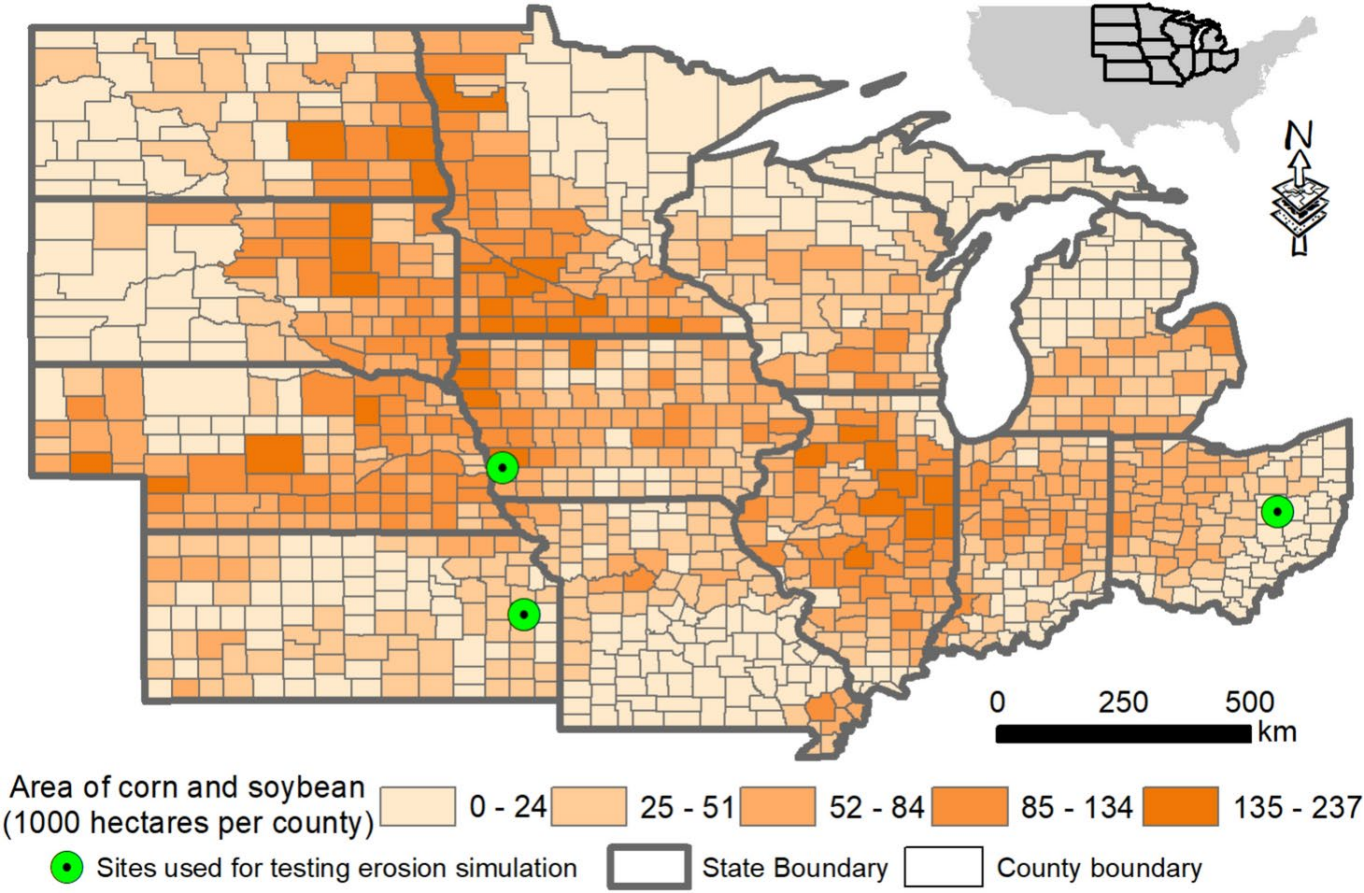
Area of corn and soybean in counties across the U.S. Midwest. The upper right inset shows the location of the U.S. Midwest within the contiguous 48 United States. Three field experimental sites (including 8 treatments) in Iowa, Ohio and Kansas were used to evaluate the EPIC model for simulating soil erosion rates of corn-soybean, CS (42.7 Mha) and continuous corn, CC (7.5 Mha) systems.

Rates of soil erosion depend on multiple factors such as soil properties, terrain, climate, and land management and are notoriously difficult to quantify.^16^ Process-based ecosystem models driven by geospatial and management data have been a powerful tool for estimating regional environmental impacts of biofuel feedstock production.^17^ Here we employ the Environmental Policy Integrated Climate—(EPIC)^18^ model (Methods and supplementary Figs. S1 and S2) to provide a conservative estimate of water erosion impacts induced by corn stover removal across 50.2 Mha cropland in the U.S. Midwest (Fig. 2). The U.S. Midwest is home to most of the biorefineries in the United States and is experiencing rapid land use change to meet human needs for food and energy^19,20^ and a significant loss of topsoil from cropland.^21^ After compiling land use, soil, climate, topography data, and a crop management database representing contemporary cropping management practices, we applied the EPIC model to simulate soil erosion in response to zero (0%), one-third (33%) and two-thirds (66%) corn stover removal in the two dominant cropping systems of the U.S. Midwest, continuous corn (CC) and corn-soybean (CS). We also assessed the effects of two different conservation scenarios: conservation practices (CP) vs. no conservation practices (NCP). The CP scenario assumes generic conservation practices are adopted to reduce soil erosion by 50% relative to the NCP scenario. The combination of different cropping systems (i.e. CC and CS), stover collection levels (i.e., 0%, 33%, and 66%), and conservation practices (CP and NCP) resulted in a total of 12 different scenarios. For each scenario, we further estimated the economic costs imposed by increased soil erosion and the accompanying lateral fluxes of soil organic carbon (SOC) using a marginal approach (Methods). By comparing with other documented impacts of corn stover-derived biofuel, we demonstrated and quantified the relevance of lateral soil and carbon fluxes for sustainable biofuel development using crop residues.

## Results

### Large increase in soil erosion rates in response to corn stover collection

**Fig. 3:**
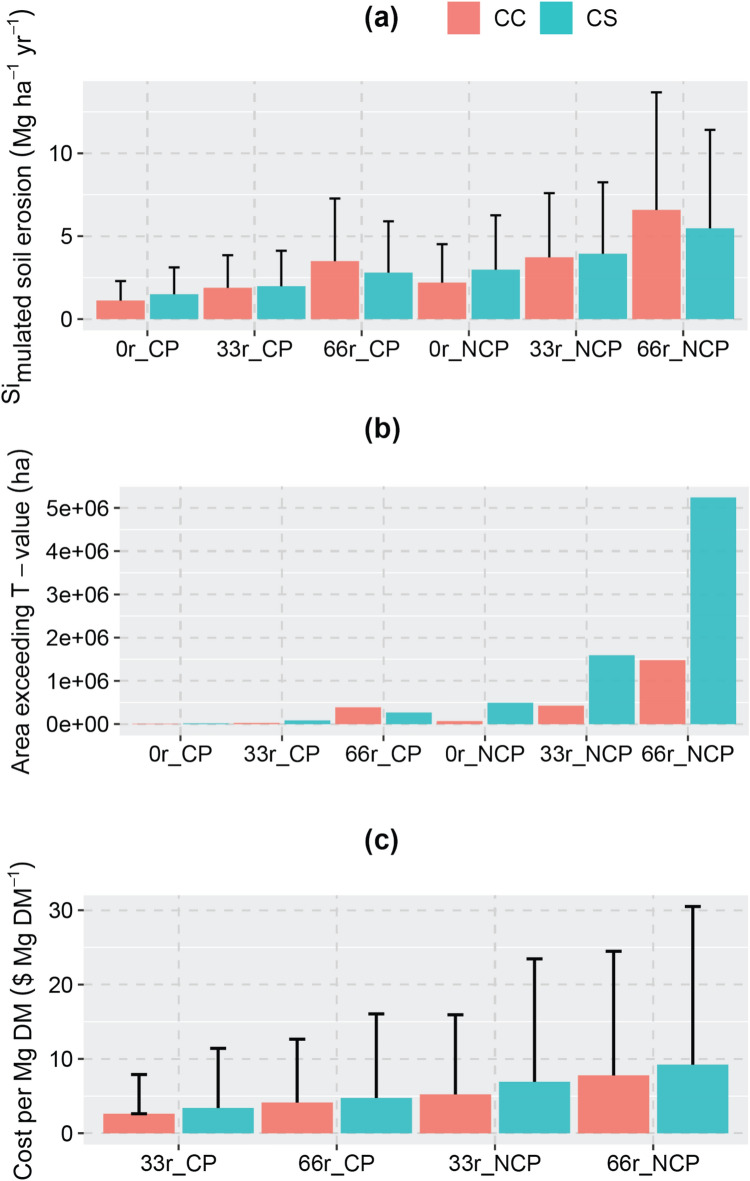
Simulated soil erosion rates averaged over the U.S. Midwest (**a**), cropland area exceeding soil erosion tolerance threshold (T-value) (**b**), and economic cost of soil erosion induced by corn stover collection (**c**) under different assumptions of soil conservations and stover removal levels. The error bars represent one standard deviation across the counties in the U.S. Midwest. CP represents a conservation scenario that reduces soil erosion by 50% as compared with the NCP scenario where no conservation practices are applied. 0r, 33r and 66r represent 0%, 33% and 66% stover removal, respectively.

Evaluated using surveyed and field experimental data, the EPIC model reliably simulated corn and soybean yields (Supplementary Figs. S3–S6) and soil erosion rates (Supplementary Fig. S7) of different CC and CS cropping systems. Regional scale simulations show that increases in stover removal intensity can result in higher soil erosion rates (Fig. 3a). We also found notable differences between the responses of soil erosion rates with respect to stover removal level for the two different cropping systems: CC and CS. With an increase in removal level from 0 to 66% under the no conservation scenario, soil erosion rates increased by a factor of 1.8, from 3.0 to 5.4 Mg ha^−1^ yr^−1^ in CS systems, and in CC systems by a factor of 3.0, from 2.2 to 6.6 Mg ha^−1^ yr^−1^. For a 33% removal level, soil erosion increased by a factor of 1.3 in CS systems and 1.7 in CC systems. With conservation practices, increased soil erosion risks were smaller. For example, soil erosion with a 66% removal level increased from 1.5 to 2.8 Mg ha^−1^ yr^−1^ for CS, and from 1.1 to 3.5 Mg ha^−1^ yr^−1^ for CC under a 66% removal level. For a 33% removal level, the soil erosion increases are 0.5 and 0.8 Mg ha^−1^ yr^−1^, respectively for CS and CC systems.

The difference in soil erosion rates between the CS and CC cropping systems interacts with the intensity of stover removal. When there was no stover removal under the conservation scenario, the soil erosion rates of CC were 26% less than those of CS. When the removal level increased to 33% soil erosion rates of CC and CS systems were comparable; and the rates increased to 25% more than CS under 66% stover removal. Similarly, under the no-conservation scenario, we found a similar pattern, where the ratio between soil erosion rates of CC and CS increased from 0.74 to 0.95, and to 1.2, respectively, as stover removal levels changed from 0 to 33%, and to 66%. Collecting corn stover has smaller impacts on soil erosion for CS than for CC.

The increases in soil erosion rates due to corn stover collection pose a threat to soil health (Fig. 3b). Under the no-conservation scenario, areas in CC with unsustainable soil erosion rates (exceeding the soil erosion tolerance threshold, T-value, of 11.2 Mg ha^−1^ ^22^) increased from 68 kha (< 1% of total CC area), to 426 kha (or 6% of total CC area) and to 1.5 Mha (or 20% of total CC area) by intensifying collection levels from 0 to 33% and 66%, respectively; Adoption of conservation practices helped to substantially reduce those CC areas to 8, 24, and 387 kha, respectively, for 0%, 33% and 66% stover collection. We observed a similar pattern for CS systems: collecting 66% stover under the conservation scenario increased the aerial extent of CS systems with unsustainable soil erosion rates from 18 to 269 kha (< 1% of the total CS area). Without conservation, the unsustainable areas increased from 490 kha to 5.2 Mha (or 12% of the total CS area).

### Soil erosion increases economic cost of corn stover collection

The increases in soil erosion due to corn stover collection, particularly from the CC system without conservation practices, could result in significant economic and long-term environmental damage. In addition to onsite effects of soil erosion, such as land degradation and fertility decline,^21,23^ excess soil erosion could also exert far-reaching offsite impacts, such as siltation and freshwater and coastal eutrophication.^24^ For example, nutrient loss from cropland in the U.S. Midwest via soil erosion and runoff is a major factor contributing to hypoxia in the Gulf of Mexico^25^ and the Great Lakes.^26^ Including both onsite and offsite impacts, Pimentel et al.^14^ estimated erosion costs were ca. $8.0 per ton of soil lost in 1992 (or $16.7 in 2022 with adjustment for inflation).

Applying the $16.7 cost per ton of sediment loss, the economic cost of collecting 33% and 66% stover from CS ranges between $6 and $36 ha^−1^ yr^−1^. For CC, the economic cost of increased soil erosion was as high as $63 ha^−1^ with 66% removal and no-conservation. This range of the economic cost of increased soil erosion ($6–$63 ha^−1^) is comparable to no-till subsidy ($50 ha^−1^), which was shown to have impacts on residue-based biofuel feedstock supply.^27^ In addition, the cost of soil erosion represents up to 20% of the net economic return of $316 ha^−1^ for conventional corn farms.^28^ When normalized by the amount of stover collected, the increased soil erosion cost for CS (ranging between $3.4–$9.2 per Mg Dry Matter (DM)) is somewhat higher than for CC (ranging between $2.6–$7.8 per Mg DM) (Fig. 3c). These numbers are comparable to costs of fuel ($3.37 per Mg DM), labor ($3.65 per Mg DM), equipment ($8.14 per Mg DM), and wrap ($7.10 per Mg DM) for stover harvesting.^29^ Compared to the estimated profitable stover price of $88.19 per Mg DM, the soil erosion cost can represent up to 10%.

### Corn stover collection causes significant amount of lateral fluxes of eroded soil organic carbon

**Fig. 4:**
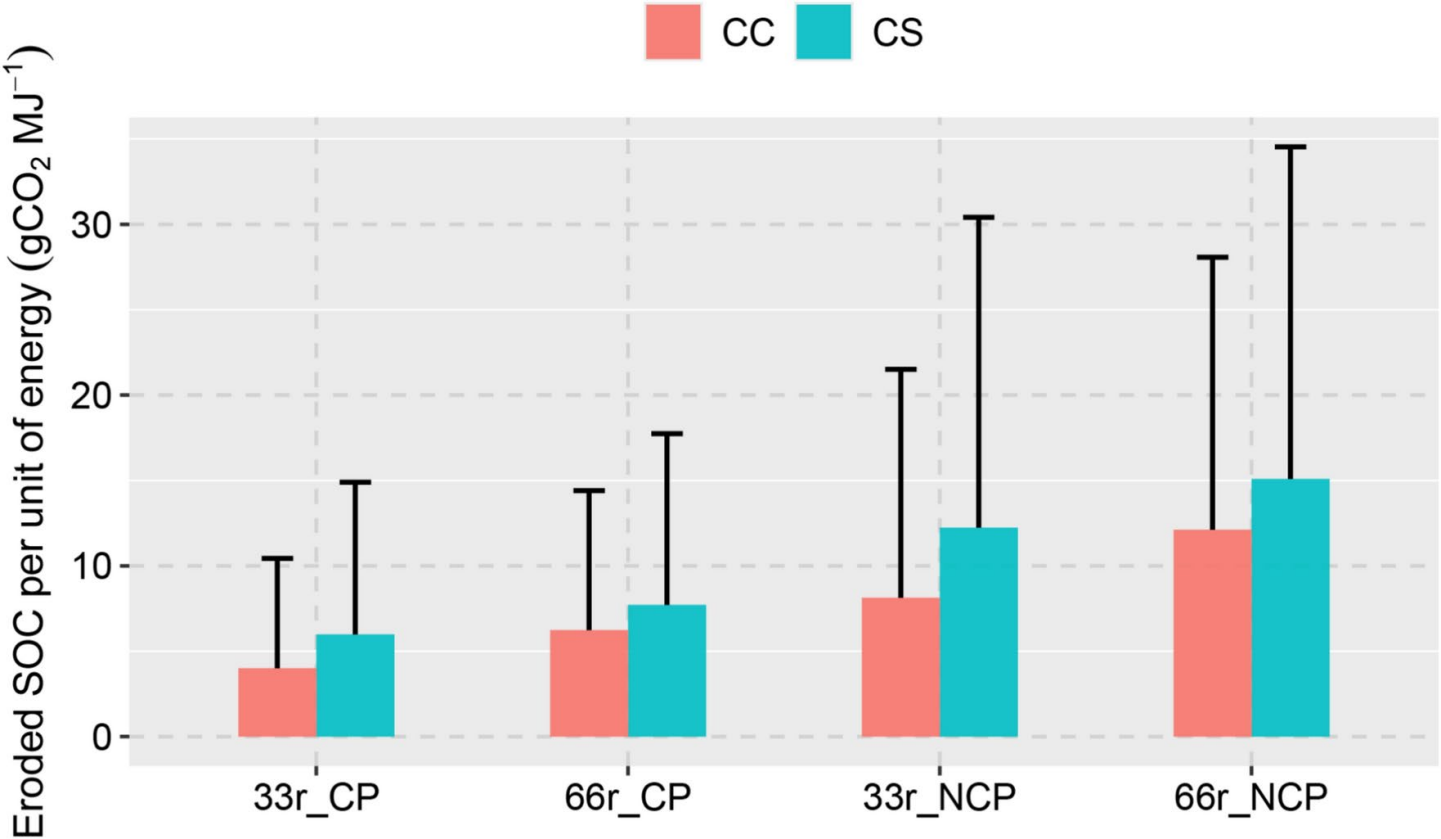
Simulated eroded SOC intensity of corn stover-derived cellulosic biofuel, averaged over the U.S. Midwest and under different assumptions of soil conservation and stover removal levels. The error bars represent one standard deviation across the counties in the U.S. Midwest.

In general, CS and CC exhibited similar amounts of eroded SOC per unit energy (Fig. 4), and higher rates of stover removal (e.g., 66% compared to 33%) led to greater amounts of eroded SOC per unit energy, because higher stover removal increased soil erosion exponentially. Adoption of conservation practices substantially reduces eroded SOC intensity. For example, eroded SOC intensity decreased from 12.5 to 6.4 gCO_2_e MJ^−1^ when implementing conservation practices for the CC system in the 66% stover collection scenario. The eroded SOC intensity, ranging between 3.9 and 12.5 gCO_2_e MJ^−1^ across all scenarios, is comparable in magnitude to other components of the life cycle impacts of the corn stover-derived cellulosic biofuel supply chain, such as distribution and combustion emissions (ca. 2.0 gCO_2_e MJ^−1^),^30^ fertilizer and diesel emissions (5–10 gCO_2_e MJ^−1^),^31^ and ca. 4.6 gCO_2_e MJ^−1^ reduction in N_2_O emissions^32^. It is also higher than the indirect land use change impacts of corn stover-based biofuel (− 0.9 gCO_2_e MJ^−1^).^33^ Note that the exact magnitude of eroded SOC intensity showed large spatial variability (as indicated by the standard deviation in Fig. 4), attributable to spatial heterogeneity in location-specific characteristics. For example, on the county level, there are 145 counties for CS and 33 counties for CC that had an eroded SOC intensity larger than 30 gCO_2_e MJ^-1^ under the no conservation and 66% corn stover removal scenario.

## Discussion

Our simulation of an average soil erosion rate of 2.9 Mg ha^−1^ yr^−1^ under the no conservation and no stover removal scenario agrees within a factor of two with a recent estimate of average soil erosion (5.2 Mg ha^−1^ yr^−1^) on cropland in the U.S. Midwest.^34^ Our lower estimates for Midwest cropping systems likely results from our allocation of intensive tillage on land with flat slopes and of low intensity tillage on highly sloped land (Supplementary Fig. S2). Such an optimal allocation, in conjunction with considering only water erosion and assuming no enrichment of SOC in eroded sediments, intended to provide a lower-end estimate of the potential soil erosion impacts of cellulosic biofuel derived from corn stover in the U.S. Even with such a conservative estimate, our regional assessment indicates that collecting corn stover for cellulosic biofuel production can substantially increase soil erosion and associated SOC loss and degrade soil health of the U.S. Midwest croplands.

Across all scenarios, 33% stover collection can increase areas with unsustainable soil erosion rates by a factor of 3–6, while 66% stover collection results in an increase by a factor of 10–50. The estimated economic cost of that increased soil erosion could significantly increase stover collection cost (by 7.5–26.5%), depending on rotation, stover collection levels, and conservation practices. With conservation practices that reduce edge-of-field soil erosion by 50%, cropping areas exceeding the soil erosion tolerance threshold could be decreased by over 70% for both CS and CC with different levels of stover collection (Supplementary Table S1). In general, collecting corn stover has smaller impacts on soil erosion for CS than for CC. This is because corn stover is only collected during corn-grown years, resulting in less amount and frequency of residue removal in CS than in CC cropping systems. Note that the soil erosion rates under the no stover removal scenarios for CC systems were smaller than that for CS systems. This is mainly because of (1) less residue left on ground from soybean than corn^35^ and (2) the difference between the average slopes of CC and CS systems, which are 2.4% and 3.0%, respectively, in this study.

In the economic cost assessment, we considered the on-site (e.g., replacement of water and nutrient loss) and off-site (e.g., siltation and water contamination) costs caused by soil erosion as reported by Pimentel et al. (1995) ^14^ with inflation adjustment. While such an approach is likely more suitable for the U.S.^14^, we also acknowledge that in other regions of the world, considering the productivity loss caused by soil erosion may be more suitable.^36^ The cost of soil erosion is comparable to that of components of the corn stover supply chain (e.g., labor and fuel costs) and a no-till subsidy that was shown to have impacts on crop residue supply, indicating the importance of factoring in the erosion cost. However, how soil erosion can influence the net income of corn farms and the selling price of corn stover deserve further research. Furthermore, the cost of conservation practices could impact the net cost of soil erosion which was not considered in this study.

The increases in eroded SOC due to stover removal (3.9–12.5 gCO_2_e MJ^−1^) are equivalent to 10.4–33.2% of the mandate of ca. 37.6 CO_2_e MJ^−1^ for cellulosic biofuel. Notably, the eroded SOC that leaves the edge-of-field can be transported to depositional landforms^37,38^ and downstream aquatic environments,^39^ where the eroded SOC can be stored or decomposed through complex physical and biogeochemical processes. There have been wide debates regarding the fate of eroded SOC as a source or sink due to the lack of consistent analysis approaches.^38^ Currently, the fraction of eroded SOC and its subsequent storage in sediment deposition sites (e.g. foot slopes, wetlands, floodplains, riverbed, and reservoirs) vs. that respired to the atmosphere, is not accounted for in the life cycle impact assessment of corn stover-derived and other types of biofuel.^40^ Although the modeling systems employed in this study estimated the amount of the water erosion leaving the crop fields, we were not able to track the fate of the eroded SOC in the downstream depositional sites. Therefore, we could not conclude that the increased eroded SOC due to corn stover removal represents a sink or source. However, the comparability of the increased eroded SOC to other components of the life cycle impacts of corn stover-derived cellulosic biofuel highlights the need of future research to elucidate the fate of eroded SOC, thereby providing more comprehensive quantification of the benefits and costs of corn stover and other crop residues derived biofuel.

**Fig. 5:**
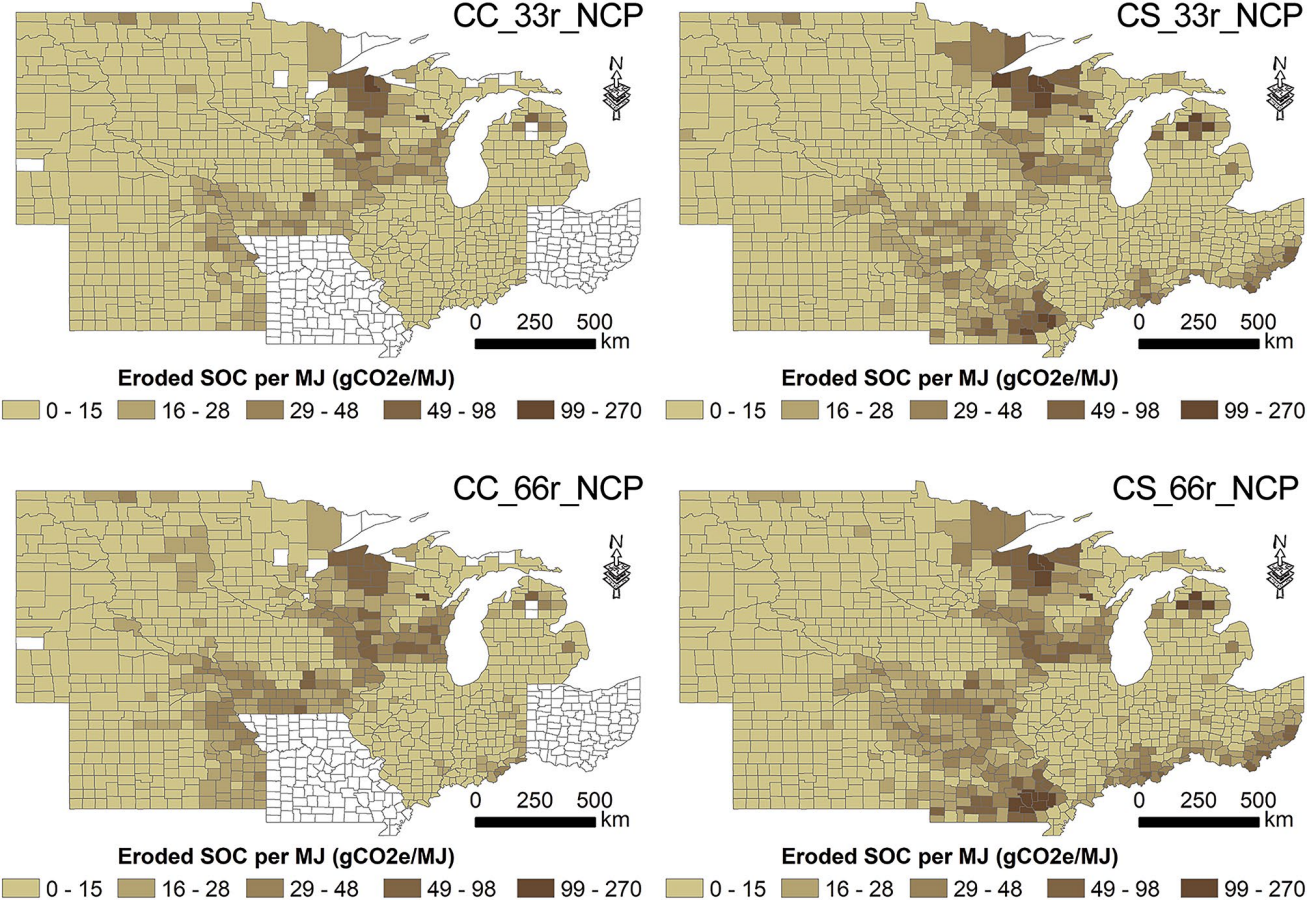
Spatial distribution of simulated eroded SOC intensity of corn stover-derived cellulosic biofuel for continuous corn (CC) and corn-soybean (CS), respectively, with 33% and 66% stover removal and no conservation practices.

Our results also show that the environmental and economic impacts of collecting corn stover vary greatly across locations (as indicated by the large standard deviation shown in Figs. 3 and 4) and between different land management scenarios (e.g., stover removal levels and conservation). In general, stover-derived cellulosic biofuel has lower eroded SOC intensity and erosion-induced economic cost from the CC system than from the CS system and adopting conservation practices hold promise to substantially reduce lateral eroded SOC fluxes. The large spatial variability, as visually illustrated in Fig. 5 for different cropping systems without conservation, show that eroded SOC intensity of corn stover-derived biofuel can vary by two orders of magnitude and is greater than other components of the life cycle impacts of cellulosic biofuel in certain areas. Therefore, the variations in soil erosion impacts on economic cost and lateral carbon fluxes between locations and crop management should be considered and quantified to optimize the corn stover supply chain to minimize negative impacts.

## Methods

### Agroecosystem modeling

We used the Geospatial Agroecosystem Modeling System (GAMS)^17^ (Supplementary Fig. S1) to integrate multiple sources of geospatial and surveyed datasets to define homogeneous spatial modeling units (HSMUs) and prepare climate, management, soils, land use, and topographic data for each HSMU (Supplementary Fig. S2) to drive the EPIC model (Supplementary Methods S1). The use of high-resolution Cropland Data Layer (CDL)^41^ and Soil Survey Geographic (SSURGO, websoilsurvey.nrcs.usda.gov) soil maps results in ca. 1 million HSMUs with CC and CS cropping systems in the U.S. Midwest. Note that the resulting HSMUs were obtained by removing numerous small areas to reduce the computational burden. Such a simplification preserved approximately 90% of crop area and skipped some cropping systems representing small fractions.^17^ For example, CC was not represented in Ohio and Missouri. Even with the reduced number of HSMUs the computational cost of executing GAMS over the study area (Supplementary Methods S3) is demanding and does not allow us to conduct exhaustive uncertainty analysis. Instead, we intended to derive conservative estimates of soil erosion and associated lateral carbon fluxes, which are unlikely to match the real-world conditions but demonstrate their potential relevance in corn-stover derived cellulosic production. Examining uncertainty of different inputs and parameters would help identify opportunities to further improve the accuracy of the estimates and quantity associated errors to support decision making. Building efficient surrogate models^42^ for the time-consuming GAMS await future research to quantify uncertainties associated with the model results. In this study, we adopted a method to allocate reported shares of different tillage practices at the county scale to each HSMU to derive a conservative estimate of water-induced soil erosion. We assumed that farmers apply no-till to steep soils and conventional tillage to flat soils and assigned reduced tillage to the remaining soils (Supplementary Fig. S2). Such an assumption maximizes the benefits of tillage for preserving soil productivity and protecting the environment. When estimating the amount of eroded SOC, we assumed no enrichment of SOC in eroded sediments as compared with the source soils, despite that enrichment ratios for eroded SOC are highly variable over time and across space. Although our previous efforts have successfully verified the EPIC model ^43,44^ for simulating eroded SOC at an agricultural site near Coshocton, Ohio,^45,46^ we could not find a method to reliably estimate the widely ranged observed enrichment ratios for eroded SOC from agricultural fields, spanning from < 0.2^47^ to 10^48^. After literature review, we found that most observed enrichment ratios for eroded SOC are larger than 1.0.^49,50^ Therefore, we chose to use 1.0 as the enrichment ratio for eroded SOC and aim to provide a conservative estimate of the magnitude of erosion-induced lateral fluxes of SOC for corn stover-derived biofuel.

### Modeling scenarios

 We implemented EPIC model runs over the U.S. Midwest corn and soybean fields from 1991 to 2050, with 1991–1999 as a warm-up period and evaluated model performance for crop yields simulation over 2000–2008. We repeated the 1991–2010 climate records twice to represent 2011–2050 and derived long-term simulations of soil erosion rates on corn and soybean fields. We explicitly simulated CC and CS cropping systems, as previous field experiments clearly indicate yield differences between corn in these two cropping systems. Model simulations were performed for two conservation scenarios (i.e. conservation vs. no-conservation). These combinations of stover removal and conservation resulted in a total of six scenarios, under which we implemented the EPIC model, respectively, for CC and CS cropping systems across the U.S. Midwest.

### Model evaluation

We evaluated EPIC model performance for simulating multi-year average corn and soybean yields using county scale US Department of Agriculture-reported data for the period 2000–2008 (Supplementary Figs. S4–S5). We also assessed the EPIC model for simulating average annual sediment yields at three locations with a total of eight treatments across the U.S. Midwest (Supplementary Figs. S7). To ensure reliable generalization of the site-scale model performance to the regional scale, we determined parameter values based on previous studies and did not conduct extensive parameter calibration to match observed variables of interest (Supplementary Methods S2). Model evaluation results indicate that the EPIC model well represents multi-year average rainfed crop yields and soil erosion rates, as well as their spatial variability.

### Model implementation

We used a Python-based parallel computing package^51^ to implement the EPIC model on the Pacific Northwest National Laboratory’s Institutional Computer (Supplementary Methods S3). For each simulation scenario, the EPIC model input–output files (ca. 8 million) were uploaded into a PostgreSQL relational database hosted on Pacific Northwest National Laboratory’s Institutional Computer to facilitate data interpretation, verification, and visualization.

### Assessment of soil erosion and lateral carbon flux impacts

We used a marginal approach^31,32^ to estimate impacts of soil erosion and lateral carbon fluxes arising from stover collection (Supplementary Eq S1). We calculated the increase in soil erosion and eroded SOC resulting from 33% or 66% stover collection by subtracting simulated values for soil erosion and eroded SOC without stover removal. Based on previous estimates of economic cost of soil erosion,^14^ we also analyzed economic impacts of soil erosion induced by corn stover collection (Supplementary Eq. 2). We multiplied the amount of soil erosion by the SOC content of the top layer of soil to estimate the potential magnitude of eroded SOC (Supplementary Eq. S3). In the calculating of eroded SOC intensity, we converted eroded SOC to CO_2_ to facilitate the (Supplementary Eq. S4) comparison with other components of the life cycle impacts of corn stover-derived biofuel. The eroded SOC intensity was normalized by the energy content of cellulosic biofuel (in MJ) derived from collected corn stover (Supplementary Eq S5).

## Supplementary Information

Supplementary Information.

## Data Availability

Contact X.Z. for the data from this study, which is available upon reasonable request.
