# Supplementary material for: Soil erosion and lateral carbon fluxes from corn stover-derived biofuel
Source: Sci Rep. 2025 May 26;15:18315. doi: 10.1038/s41598-025-99218-y (PMC12106714; doi:10.1038/s41598-025-99218-y)
Supplement: Supplementary file 1 — Supplementary Information. [file 41598_2025_99218_MOESM1_ESM.docx]

Supplementary Information for:

**Soil erosion and lateral carbon fluxes from corn stover-derived biofuel**

Xuesong Zhang^1^*, Stephen D. LeDuc^2^, Seungdo Kim^3,4^, Bruce E. Dale^3,4^, Kaiguang Zhao^5^, Yuyu Zhou^6^, Gregory W. McCarty^1^, Glenn E. Moglen^7^

^1^ USDA-ARS Hydrology and Remote Sensing Laboratory, Beltsville, MD 20705-2350, USA.

^2^ U.S. Environmental Protection Agency, Office of Research and Development- National Center for Environmental Assessment, 109 TW Alexander Dr., Research Triangle Park, NC 22709, USA.

^3^ Great Lakes Bioenergy Research Center, Michigan State University, East Lansing, MI 48824, USA.

^4^ Chemical Engineering and Materials Science, Michigan State University, 3815 Technology Boulevard, Lansing, MI 48910, USA.

^5^ Environmental Science Graduate Program, School of Environment and Natural Resources, The Ohio State University, Columbus, OH 43210, USA.

^6^ Department of Geological and Atmospheric Sciences, Iowa State University, Ames, IA 50011, USA.

^7^ Department of Civil and Environmental Engineering, The University of North Carolina at Charlotte, Charlotte, NC 28223, USA

**This file includes:**

Supplementary Methods: 1–3

Supplementary Figures: 1–7

Supplementary Equations: 1–5

Supplementary Tables: 1

50e_00r_cs

50e_66r_cs

Supplementary
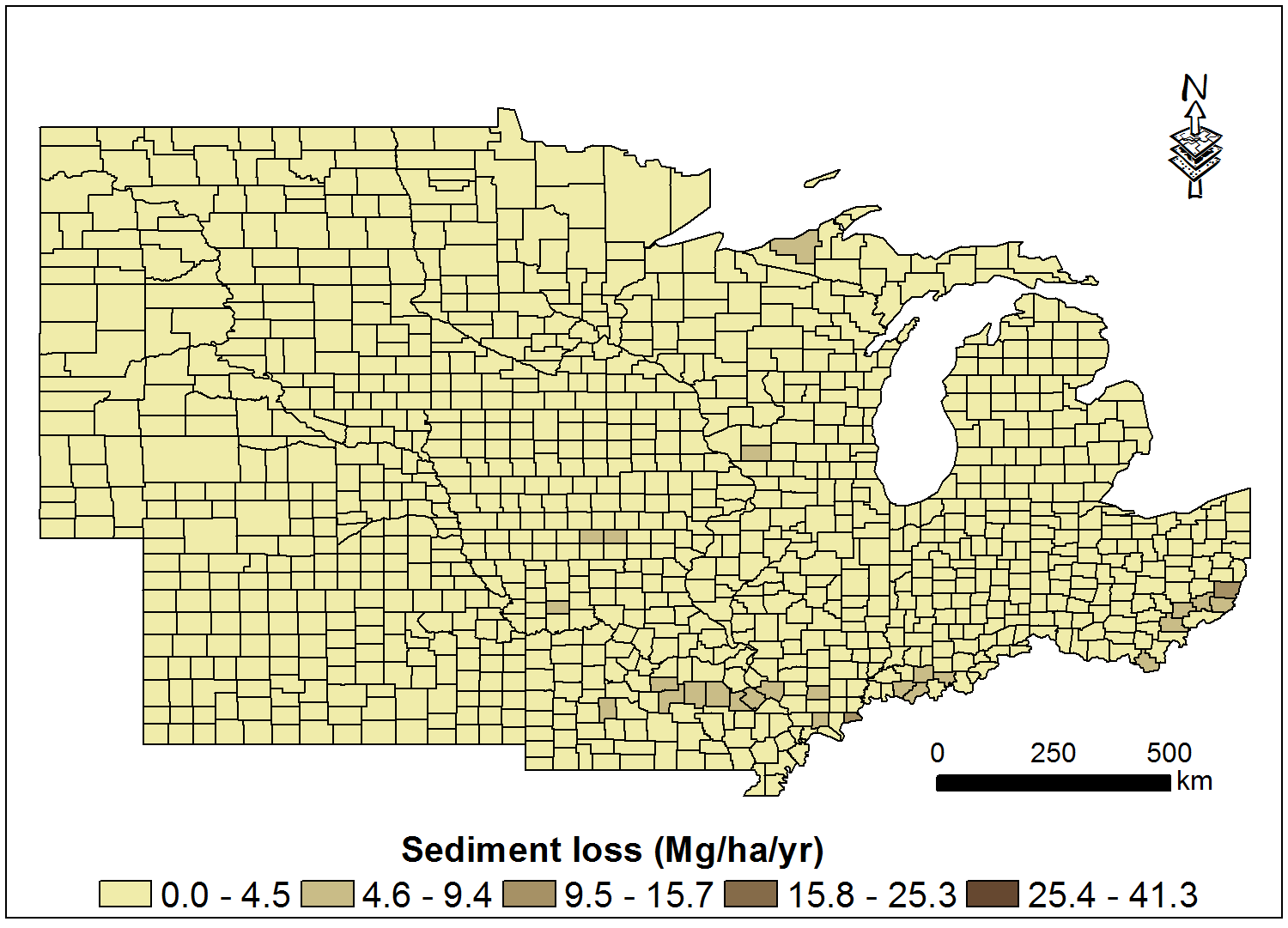
References: 1–55

50e_00r_cs

50e_66r_cs

# Supplementary Materials - Methods

## S1 Description of the EPIC Model

The Erosion Productivity Impact Calculator (EPIC) model^1,2^ is an agroecosystem model that has been widely tested for numerous locations and cropping systems across the globe and used in understanding agronomic and environmental impacts of agricultural management ^3^. The EPIC model uses a revised version of Crop Environment REsource Synthesis (CERES)^2,4^ to simulate plant growth, and considers effects from vapor pressure deficits, atmospheric CO_2_ concentrations, nutrients availability, and other environmental controls and stresses. The EPIC model’s hydrology module contains terrestrial water cycling processes including snowmelt, surface runoff, infiltration, soil water content, percolation, lateral flow, water table dynamics, and evapotranspiration. EPIC uses the Modified Universal Soil Loss Equation (MUSLE)^5,6^ Currently, the EPIC model is parameterized for approximately 120 plant species including food crops, native grasses, and trees. The EPIC model has been extensively tested for many agricultural cropping systems, and applied to understand agronomic and environmental impacts of alternative management practices ^3^. The crop growth and soil organic carbon modules of EPIC have been examined against field observations from numerous sites in the US Midwest and across the world ^7-21^.

## S2 Model Parameterization

We obtained typical planting and harvesting dates for corn soybean from the USDA surveyed data (<https://usda.library.cornell.edu/concern/publications/vm40xr56k?locale=en>). Annual nitrogen and phosphorus fertilizer application rates were estimated based on the state-level statistics from USDA-ERS (<https://www.ers.usda.gov/data-products/fertilizer-use-and-price.aspx>). At the county level, we derived the fractions of tillage practices compiled by the Conservation Technology Information Center (<https://www.ctic.org/CRM>), which were grouped into three categories: conventional tillage, reduced tillage, and no-till and gap-filled for 2000-2008 ^22^. We allocated different tillage practices to each HSMU by assuming that farmers apply no-till to steep soils to preserve soil productivity and protect the environment. It is worth noting that we do not have extensive evidence showing that such an assumption matches farmers’ decision making. Therefore, this assumption is only intended for the purpose of providing conservative estimates of soil erosion and associated carbon fluxes, instead of matching reality. Therefore, future research on uncertainty analysis would help better understand the sensitivity of different inputs and identify opportunities to further improve the estimates.

Conventional tillage was assigned to flattest HSMUs, while no-till was applied to HSMUs with steepest slopes. The remaining HSMUs implemented reduced tillage. For each HSMU, we further derived climate and management information to drive EPIC model runs. First, we located for each HSMU the closed climatological grid of North-American Land Data Assimilation System 2 (NLDAS-2; ldas.gsfc.nasa.gov/nldas/), which contains climate data (temperature, precipitation, solar radiation, wind speed, and relative humidity).

Key parameters and initial state variables need to be determined before running models. One parameterization strategy that has been adopted in model assessment ^18,23^ consists in parameterizing variables based on prior information (e.g. from literature or field experiments) ^3,7,15,16,20,24,25^ without attempting to extensively calibrate parameters to match observed variables of interest. In that case, model performance is highly dependent on the quality of input data, instead of the parameter values obtained through calibrating the model to fit with the data at one or multiple fields. Such a parameterization scheme helps realistically generalize the site-scale model performance to regional scale.

The continuous development of spatial data for climate, terrain, crop classification, and soils has resulted in dramatic increase in spatially-explicit information and provided new opportunities to further advance the application of conceptual models. In this study, we did not modify the crop, hydrologic, and biogeochemical parameters within the EPIC model that have been extensively tested in previous studies ^3,7,15,16,20,21,24-26^, but focused on deriving data-based agroecosystem parameters to characterize cropping systems and drive the EPIC model, as discussed in the above section on compilation of geospatial and multi-scale surveyed data.

Note that, as most management data, such as tillage and fertilization, are not available in a spatially explicit way, our aim was not to achieve accurate simulation of soil erosion at the field scale, but intend to represent a lower end estimate of the soil erosion rates and associated impacts at coarser scales (e.g. county level). We implemented the EPIC model over the U.S. Midwest corn and soybean planting areas from 1991-2050, with 1991–1999 as a warm-up period and evaluated model performance for crop yields simulation over 2000–2008. We repeated the 1991–2010 climate records twice to represent 2011–2050 and derived long-term simulations of soil erosion rates on corn and soybean fields. Capped by the attainable corn stover removal level less than 75% reported previous studies ^27,28^, we simulated three levels of stover removal (i.e. 0%, 33% and 66%). As tillage mixes surface residue into soils, increasing the period between harvesting and tillage could extend the protection of surface soils and reduce erosion.

## S3 Parallelized Model Execution

For the entire U.S. Midwest, serially executing the EPIC model over 1 million HSMUs over the period of 1991-2050 EPIC model runs would require ca. 2000 hours or 83 days. That is enormously time consuming and necessitates the use of parallel computing on supercomputers to improve modeling efficiency. We modified the Python-based software package developed by Nicolas et al. (2012) and Zhang et al. (2013)^29,30^ to implement EPIC model runs in parallel. We combined Python (python.org), mpi4py ^31^ and OpenMPI ([www.open-mpi.org](http://www.open-mpi.org)) to make use of dozens of processors simultaneously. As illustrated in Supplementary Figure 1, the software first identifies a Master processor and splits the entire set of EPIC model runs into a specified number of folders. Next, the Master processor sends commands to a few processors to execute the EPIC model in each of the folders in parallel. The parallel computing package also extracts, organizes, and uploads EPIC model-simulated agronomic and environmental variables stored in millions of text files into a PostgreSQL relational database to facilitate data query, analysis, and visualization. With this parallelization strategy, we reduced the amount of time required to complete the EPIC model runs for one scenario from 83 days to about 18-20 days (dependent on the workload currently running on the supercomputer). A major reason for not being able to fully utilize the parallel computing power is that each EPIC model run needs to read/write 10 files and simultaneously executing multiple EPIC model runs would involve numerous I/O operations, lowering the efficiency.

# Supplementary Materials – Figures


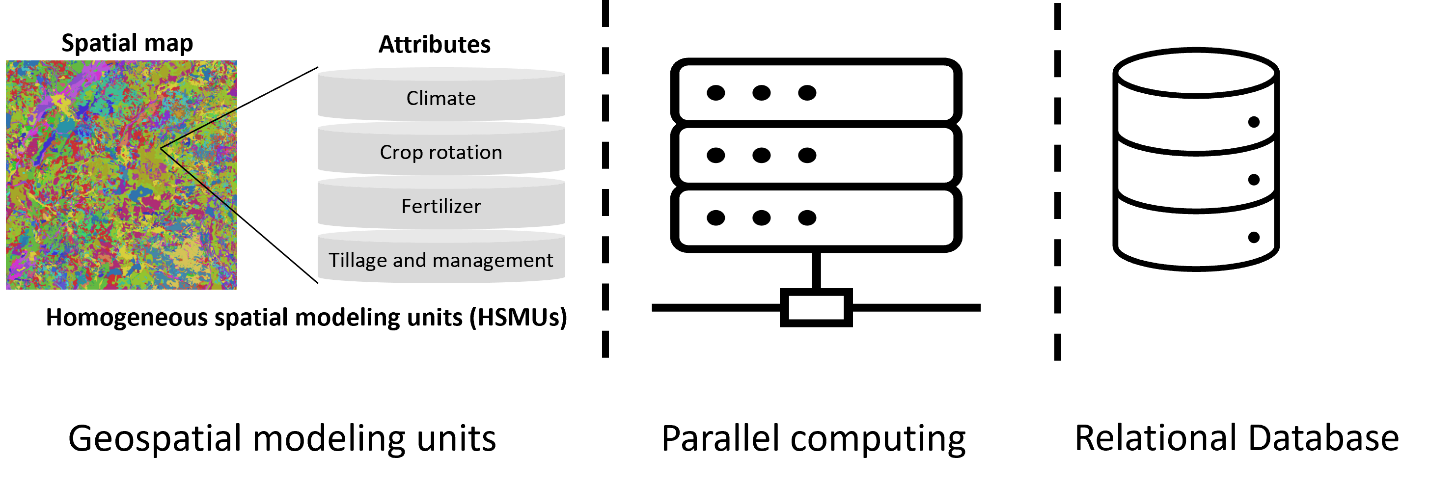


## Supplementary Figure S1.

Overview of regional modeling framework. This figure is revised based on Zhang et al. (2015) ^26^. We used the Geospatial Agroecosystem Modeling System (GAMS) to integrate multiple sources of geospatial and surveyed datasets to define homogeneous spatial modeling units (HSMUs) and prepare relevant climate, management, soils, land use, and topographic data for each HSMU to drive EPIC modeling. The use of high-resolution Cropland Data Layer (CDL) and Soil Survey Geographic Database (SSURGO) soil maps results in ca. 1 million HSMUs with continuous corn (CC) and corn-soybean (CS) rotations, and ca. 8 million input-output files for EPIC model implementation. GAMS uses the Python-based parallel computing package ^29^, which binds Python3.0 and mpi4py, to run the EPIC model in parallel for the 1 million HSMUs on the Pacific Northwest National Laboratory’s (PNNL) Institutional Computer (PIC). The third component of GAMS is Python-based post-processing software that extracts variables of interest from the EPIC model input-output files and upload them into a PostgreSQL relational database, which allows to query and organize the data to facilitate interpretation, verification, and visualization.


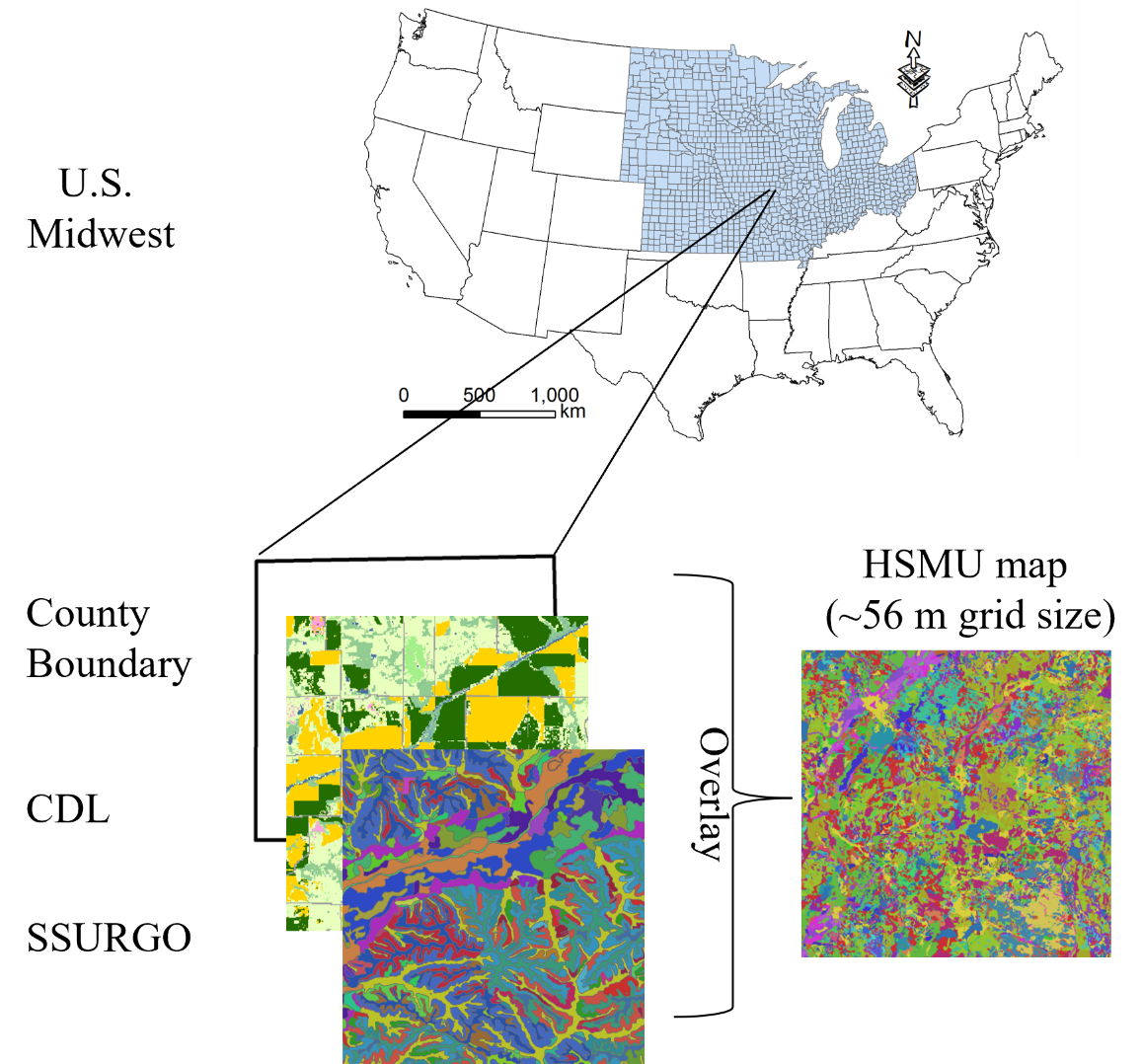


## Supplementary Figure S2.

Schematic illustration of procedures for defining HSMUs, and the flexible hierarchical organization of geospatial data.

We compiled a series of geospatial databases, including land use / land cover, soil, catchment and geopolitical boundaries, and topography data, to define HSMUs and provide relevant parameters to drive the EPIC model. **Crop rotation map**. We combined four years of Crop Data Layer (CDL) data from 2007–2010s and identified crop rotations for the U.S. Midwest^32^. **Soils.** We converted the SSURGO maps (websoilsurvey.nrcs.usda.gov) into a raster format with a resolution of 30 m, in alignment with CDL. We also processed soil properties data for the EPIC model that included number of soil layers, layer depth, slope gradient and length, albedo, bulk density, pH, percent sand, silt, clay and coarse fragments, and soil organic matter. **Elevation**. We use the Shuttle Radar Topography Mission (SRTM) digital elevation model (DEM)^33^ to estimate elevation of each HSMU to calculate atmospheric pressure. **Geopolitical Boundaries**. County and state boundaries were also used to define HSMUs. This allows us to link the surveyed crop management data at the state and county levels with HSMUs.

By overlaying the geospatial layers, we obtained HSMUs with unique properties defined by the following dimensions: unique ID, latitude, longitude, elevation, slope, crop rotation, soil type, county, state, and hydrologic unit^25^. The spatially explicit scheme employed here resulted in millions of modeling units, preventing us from conducting analysis of numerous scenarios. On the other hand, such a scheme allows us to flexibly configure county scale tillage share data to the HSMU level to represent a scenario with conservative estimates of soil erosion, and summarize HSMU level results into multiple scales (e.g. site, county or state level) to compare with observations or surveyed data for model evaluation and reporting.


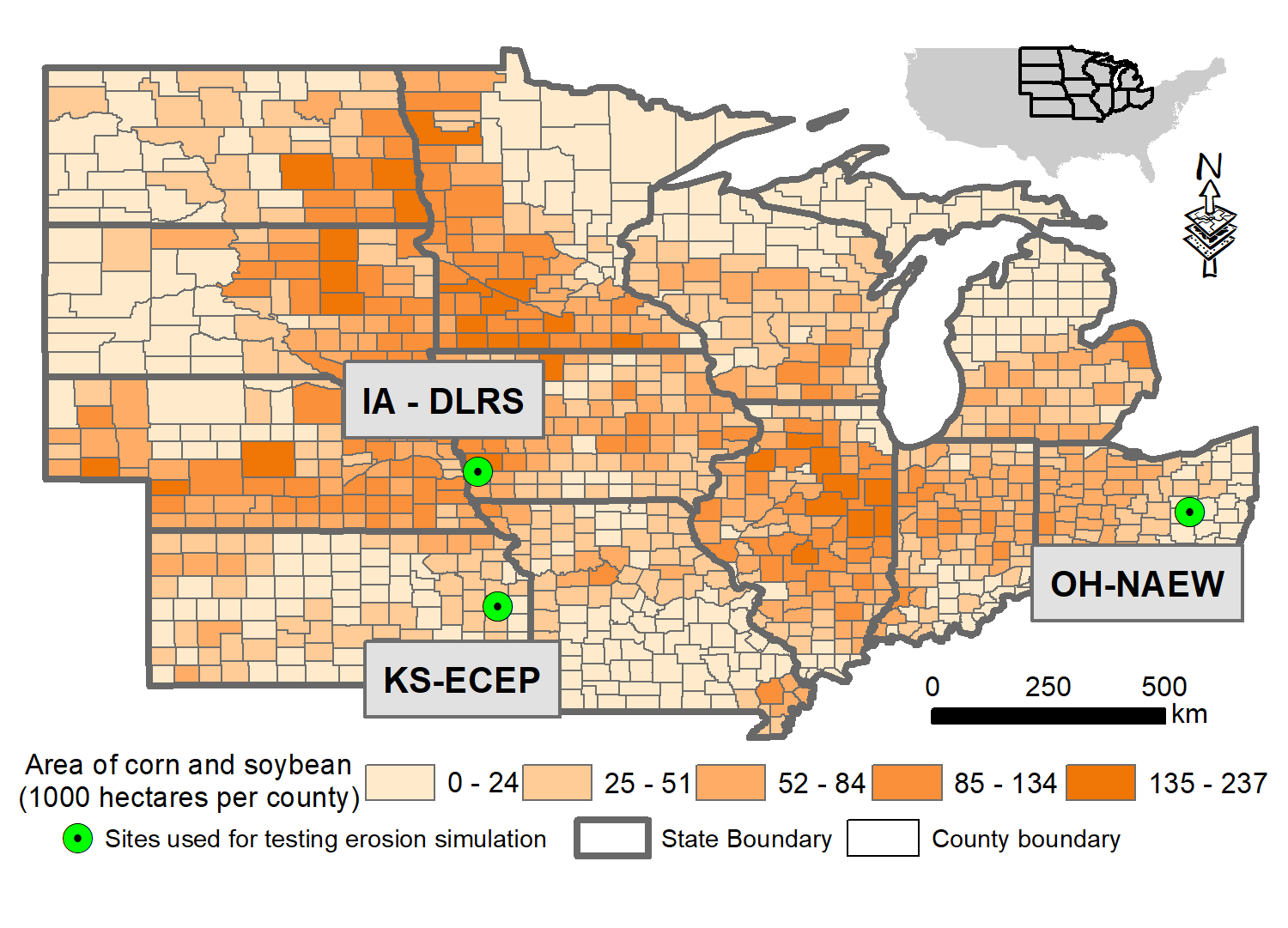


## Supplementary Figure S3.

Location of the experimental sites used to evaluate performance of the EPIC model for simulating soil erosion.

The USDA’s North Appalachian Experimental Watershed (NAEW) research station (40°22’ N, 81°48’ W) near Coshocton, Ohio hosts long-term agricultural field experiments^34^. Here we used the measured soil erosion data from the W118 experimental site (0.79 ha) to evaluate the EPIC model. Within the long history of measurements, we selected two treatments: (1) conventionally tilled corn between 1971 and 1975 and (2) no-till corn-soybean during 1984–1998. Contouring planting is adopted on the field. More details about the W118 experimental site are available in Owens et al. (2010)^34^ and Hao et al. (2001)^35^. The two treatments used here within W118 are named as OH-CC-CT-W118 and OH-CC-NT-W118, respectively.

The soil erosion test datasets also include two experimental fields (i.e. W2 and W3) of the USDA National Soil Tilth Laboratory’s Deep Loess Research Station (DLRS) ^36^, which are located near the town of Treynor in Pottawattamie County, Iowa. The W2 site with an area of 34.4 ha was under conventionally tilled continuous corn, while W3 with an area of 43.3 ha was ridge-tilled continuous corn. For both sites, contour farming was applied. More details about the characteristics of the two sites are available in Chung et al. (1999) ^37^ and Wang et al. (2008) ^24^.The averaged soil erosion rates for 1976-1995 were 12.3 and 1.5 Mg ha^-1^ yr^-1^, respectively for W2 and W3. As the major difference between these two sites is the tillage intensity, the results clearly illustrate that increasing tillage intensity sharply increases soil erosion rates. Therefore, our strategy of assigning no-till or conservation tillage practices to deeply sloped fields and conventional tillage to gently sloped land represents an optimal scenario for reducing soil erosion. That is a major reason that our estimates of soil erosion rates from cropland in the U.S. Midwest are lower than other estimates. The two experimental fields are denoted as IA-CC-CT-W2 and IA-CC-RT-W3, respectively.

In addition, the (Kansas State University) KSU-East Central Experiment Field (ECEP) (38°32′N, 95°15′W) in Ottawa with different treatments (6 m by 6 m plots) of residue removal ^38^ was used here to assess the EPIC model to simulate effects of corn stover on soil erosion rates. The stover removal levels include 0%, 25%, 50% and 75% from rainfed, no-till, and continuous corn fields, which envelope the 33% and 66% stover removal scenarios examined in our regional assessment. The four residue removal treatments are represented as KS-CC-NT-NoRemoval, KS-CC-NT-25%removal, KS-CC-NT-50%removal, and KS-CC-NT-75%removal, respectively.

For W2 and W3, runoff volume was measured using a broad-crested, V-notch weir instrumented at the outlet of each watershed ^24,39^ and water samples were collected 50-125 m above the weirs that represent sediment concentration from overland flow. At 118, runoff volume was measured using a H-flume and water stage recorder and sediment concentration in runoff was measured by filtration of 10 cm^3^ of runoff water ^35^. At W2, W3 and W118, the water erosion is estimated by multiplying sediment concentration with total runoff leaving the watershed. In contrast, at the KSU-East Central Experiment Field, sediment loss was measured based on rainfall simulation rained on 2.5 m^2^ runoff subplots within the main plots, and water samples were collected through a V-shaped runoff collector. It is worth noting these experimental watersheds/plots used various methods for measuring runoff and sediment load. Such differences could introduce uncertainties in observed data, for which there is a lack of information to quantify. Therefore, the model assessment against those observed data generally reflects that the model can capture the general patterns of water erosion under different residue removal intensity but should not be interpreted as the accuracy that the model captures in real-world conditions.


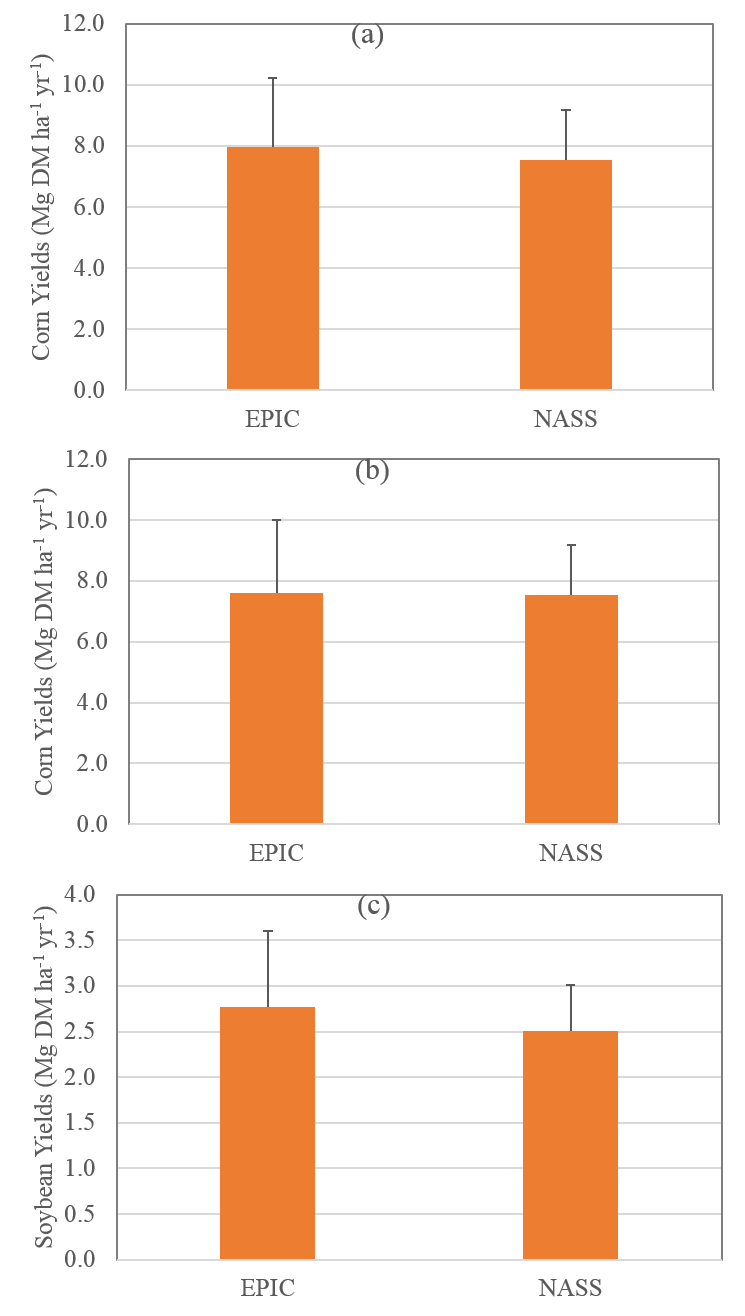


## Supplementary Figure S4.

Comparison between multi-year (2000–2008) average corn and soybean yields simulated by the EPIC model and reported by USDA NASS on a dry matter (DM) basis: (a) corn yields in CS, (b) corn yields in CC, and (c) soybean yields in CS.

We conducted model evaluation for corn and soybean, respectively, by comparing EPIC model simulations with those reported by USDA National Agricultural Statistics Service (NASS) averaged over 2000–2008 at the county level. Note that, we did not include irrigation in EPIC model simulations due to the lack of detailed information about irrigation practices. When calculating average corn and soybean yields, we excluded Nebraska and Kansas due to their extensive irrigation practices. Therefore, our model simulations represent crop yields and soil erosion under rainfed conditions, and caution should be exercised for model simulations in the two intensively irrigated states. As NASS does not differentiate corn yields in CC and CS, we could not directly verify EPIC model performance under these two cropping systems at the regional scale. Here we identified those counties that have both EPIC model simulated corn yields under CS and CC and NASS reported lumped corn yields to assess EPIC model performance. In general, EPIC captured the regional average corn yields well, with biases less than 7% for both CC and CS. Corn yields in CC are on average about 5% lower than those in CS, which fall well within the range of the differences between corn yields in CC and CS^40,41^. As to soybean yields, EPIC attained an overestimate by 12% as compared against NASS. Overall, the EPIC model simulated well the magnitude of average corn yields under both CC and CS and soybean yields under CS.


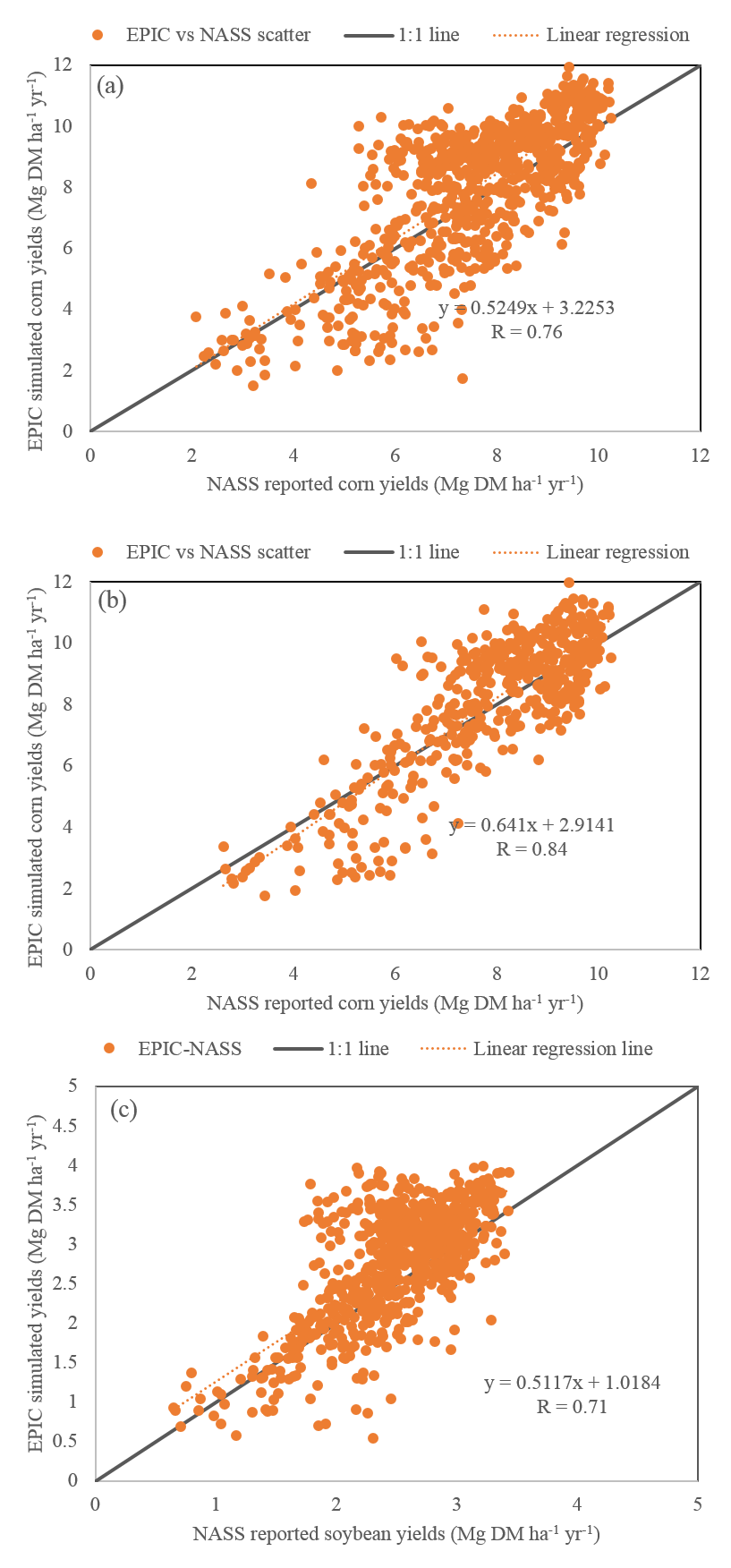


## Supplementary Figure S5.

Scatter plot of EPIC model simulated, and USDA NASS reported county level multi-year (2000–2008) average corn and soybean yields: (a) corn yields with CS, (b) corn yields with CC, and (c) soybean yields with CS. Like Supplementary Figure 3, we did not include Nebraska and Kansas in this comparison. The EPIC model simulated corn yields under both CS and CC correlated well with those reported by NASS (as indicated by correlation coefficient (R) of 0.76 and 0.84, respectively). For soybean yields, the EPIC model also explains a large fraction of the variability in NASS reported county yields with a R value of 0.71.

## Supplementary Figure S6.


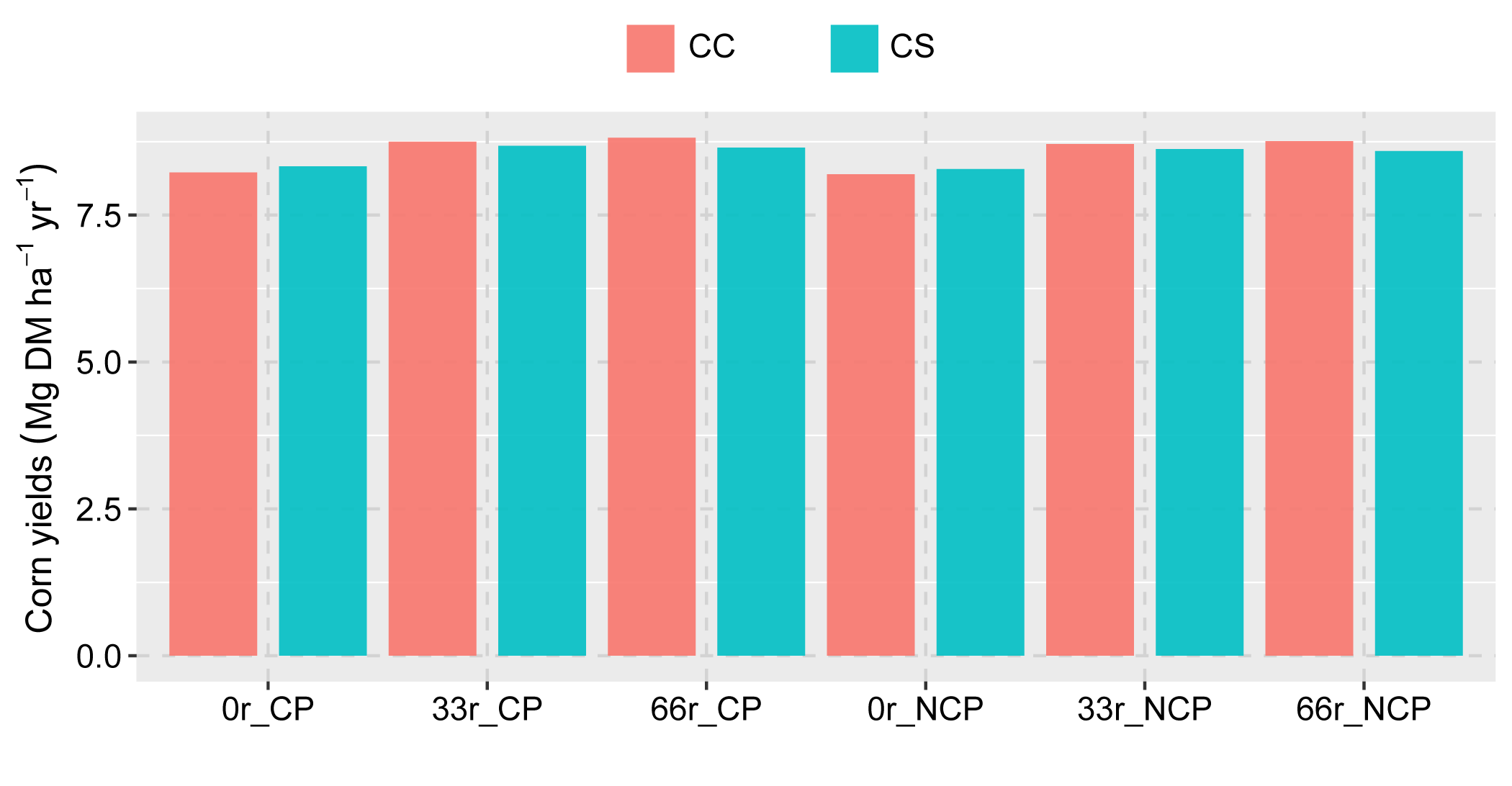


Corn yields in CC and CS as influenced by different conservation and stover removal levels. The scenarios with conservation practices (CP) assumes generic conservation practices are applied to reduce soil erosion by 50% as compared with the scenarios where no conservation practices (NCP). 0r, 33r, and 66r represent no stover removal, 33% removal, and 66% removal, respectively.

Quantification of erosion induced changes in crop yields has been a long lasting challenge^42^. Previous studies ^43-45^ reported mixed positive and negative effects of soil erosion on crop productivity dependent on the interplay between numerous factors (such as soil properties, climate, crop types, and management practices), but concluded that increased soil erosion in general reduces crop yields. We simulated slightly lower (less than 1%) corn yields in non-conservation scenarios than in the conservation scenarios under the same rotation and residue removal level, which is generally in agreement with previous findings^45^. The effects of stover removal on corn yields could be positive ^38,46^, negative ^47,48^, or neutral ^49,50^, and are difficult to determine^42^. Data reported in previous studies show that less than 50% residue removal could increase crop yield and residue inputs ^38,51-54^. For example, Battaglia et al. reviewed the impacts of the agronomic impacts of stover removal, and found that in most cases stover removal has little or even modestly positive impacts on crop yield, except for water limited conditions^54^. Therefore, the simulated slight increases in crop yield under stover removal is in general in line with the field observations.


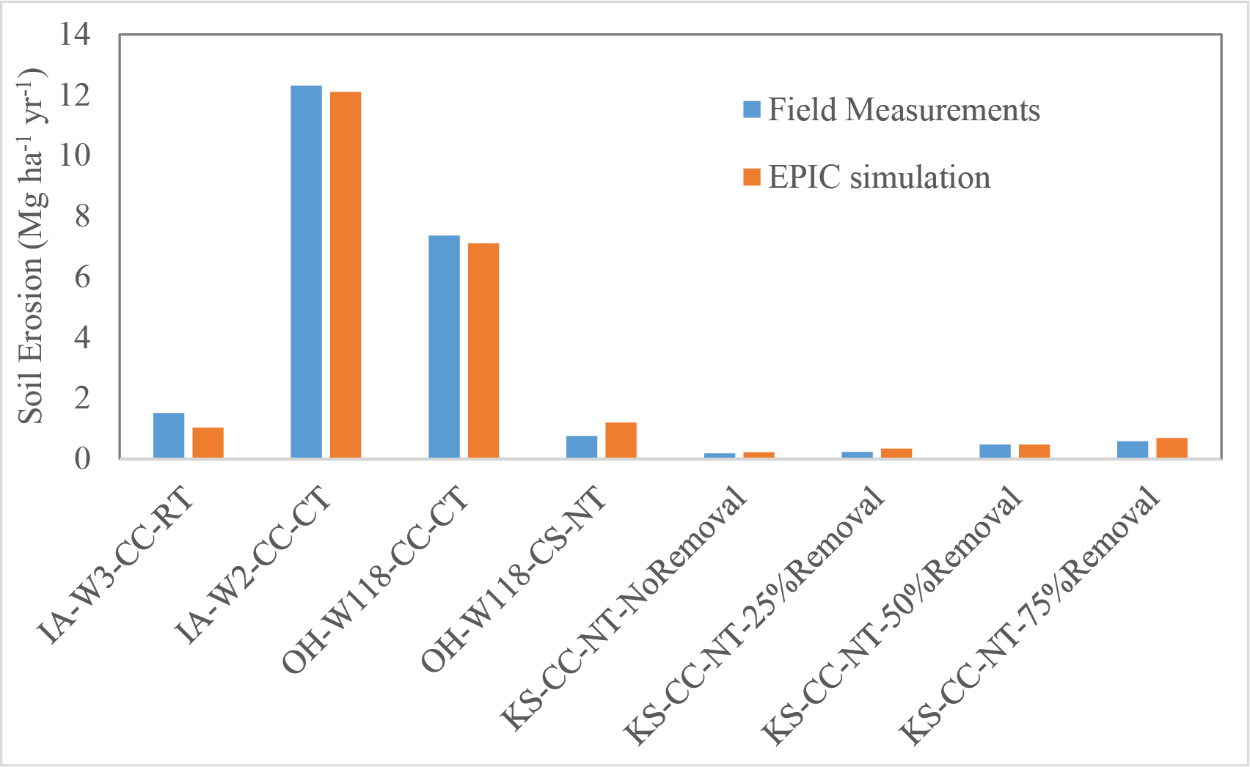


## Supplementary Figure S7.

Measured and simulated soil erosion rates at the eight experimental treatments. The field measurement data represent the average annual values of the observational years. The EPIC model simulation is the 50-year average between 2001 and 2050, with the 2011–2050 climate condition represented by repeating the climate records of 1991–2010. The EPIC modeling site was selected as the HSMU to be collocated with and has the same soil type as an experimental field. We further modified the HSMU’s crop management, including rotation, tillage intensity, fertilization, and conservation practices to reflect the management information reported in the literature. Deviation of EPIC model simulations from observations ranged between -32.45% and 59.72% across the eight experimental treatments. For the four treatments with residue removal in Kansas, the EPIC model reasonably estimated the increase of soil erosion as the result of increases in removal. For example, observed soil erosion increases of 0.05 Mg ha^-1^ yr^-1^ (with removal from 0% to 25%), 0.29 Mg ha^-1^ yr^-1^ (with removal from 0% to 50%), and 0.41 Mg ha^-1^ yr^-1^ (with removal from 0% to 75%), are in general comparable to EPIC simulated corresponding soil erosion increases of 0.12 Mg ha^-1^ yr^-1^, 0.26 Mg ha^-1^ yr^-1^, and 0.45 Mg ha^-1^ yr^-1^.

# Supplementary Materials – Equations

## Supplementary Eq S1: A marginal approach used to calculate soil erosion impacts of stover collection

In calculating the impacts of corn stover removal on soil erosion, we used the marginal approach that has been used in assessing sustainability of corn residue derived cellulosic biofuel (^28,55^). Soil Erosion Rate (SER) changes due to corn stover removal (ΔSER, Mg ha^-1^ yr^-1^) are estimated with the following equation:

${\Delta SER}_{xx\%,conservation}\text{=}{SER}_{xx\%,conservation}- {SER}_{0\%,conservation}$ (Eq 2)

where ${\Delta SER}_{xx\%,conservation}$ represent the marginal impact of xx% (i.e. 33% or 66%) stover removal under the conservation scenario on soil erosion rates. Similar equations can be used to calculate ${\Delta SER}_{xx\%,no-conservation}$ under the no-conservation scenario.

## Supplementary Eq S2: Economic cost of soil erosion

$\mathrm{Cost}_{\Delta\mathrm{SER}}\text{=}\Delta SER\times E$ (Eq 3)

where $\mathrm{Cost}_{\Delta\mathrm{SER}}$ represents the marginal impact of stover collection on economic cost ($), $E$ is the economic cost of soil erosion (in the unit of $ Mg sediment^-1^).

## Supplementary Eq S3: Eroded SOC intensity calculation

$\mathrm{SOC}_{\Delta\mathrm{SER}}\text{=}\Delta SER\times{SOC}_{content}\times100$ (Eq 3)

where $\mathrm{SOC}_{\Delta\mathrm{SER}}$ is eroded SOC (in the unit of Mg C ha^-1^ yr^-1^) as induced by stover collection, and ${SOC}_{content}$ is the SOC content of topsoil (%).

## Supplementary Eq S4: CO_2_e of eroded SOC

$\mathrm{CO}_{2}e \left( g \mathrm{CO}_{2} \right)={SOC}_{eroded}\times Mg C\times\frac{44 Mg \mathrm{CO}_{2}}{12 Mg C}\times\frac{{10}^{6} g \mathrm{CO}_{2}}{1 Mg \mathrm{CO}_{2}}$ (Eq 4)

where: ${SOC}_{eroded}$ is the amount of eroded SOC (in Mg C). The conversion from C to CO_2_ is based on their atomic weights.

## Supplementary Eq S5: Energy equivalent of dry biomass

$Energy Equivalent (MJ)=DM\times\mathrm{Conversion}_{\mathrm{efficiency}}\times\frac{Gallon Fuel}{Mg DM}\times\mathrm{Biofuel}_{\mathrm{energy}}$ (Eq 1)

where DM is dry mass of biomass yield (Mg ha^-1^ yr^-1^), $\mathrm{Conversion}_{\mathrm{efficiency}}$ is the conversion factor for biofuel production from corn stover (75 Gallons per Mg DM), and $\mathrm{Biofuel}_{\mathrm{energy}}$ is biofuel energy content (80 MJ Gallon^-1^)^13,55^.

The ΔSER, $\mathrm{Cost}_{\Delta\mathrm{SER}}$, and $\mathrm{SOC}_{\Delta\mathrm{SER}}$ can be normalized by the amount of stover (in the unit of DM Mg) collected or the amount of corn stover-derived cellulosic biofuel (in the unit of MJ) for comparison purposes. The marginal approach helps reflect the environmental impacts that the increases in stover removal intensity are responsible for.

# Supplementary Materials – Tables

## Supplementary Table S1

Effects of conservation practices on cropland areas exceeding soil erosion tolerance threshold. CP represents a conservation scenario that reduce soil erosion by 50% as compared with the NCP scenario where no conservation practices are applied

| Cropping system | Stover removal | Area with NCP (ha) | Area with CP (ha) | Reduction due to conservation (%) |
| --- | --- | --- | --- | --- |
| CS | 0% | 492,759 | 17,540 | -96% |
|  | 33% | 1,592,965 | 85,381 | -95% |
|  | 66% | 5,243,039 | 26,8571 | -95% |
| CC | 0% | 67,798 | 7,670 | -89% |
|  | 33% | 425,620 | 23,755 | -94% |
|  | 66% | 1,477,422 | 386,614 | -74% |

# Supplementary References

50e_00r_cs

50e_66r_cs

1 Williams, J., Jones, C., Kiniry, J. & Spanel, D. A. The EPIC crop growth model. *Transactions of the ASAE* **32**, 497-0511 (1989).

2 Jones, C. *et al.* EPIC: an operational model for evaluation of agricultural sustainability. *Agricultural Systems* **37**, 341-350 (1991).

3 Wang, X. *et al.* EPIC and APEX: Model use, calibration, and validation. *Transactions of the ASABE* **55**, 1447-1462 (2012).

4 Williams, J., Jones, C., Kiniry, J. & Spanel, D. A. The EPIC crop growth model. *Transactions of the ASAE (USA)* (1989).

5 Wischmeier, W. H. & Smith, D. D. Predicting rainfall erosion losses-a guide to conservation planning. *Predicting rainfall erosion losses-a guide to conservation planning.* (1978).

6 Williams, J. Sediment routing for agricultural watersheds 1. *JAWRA Journal of the American Water Resources Association* **11**, 965-974 (1975).

7 Aklesso, E.-M., Swinton, S., Izaurralde, R. C., Manowitz, D. H. & Zhang, X. Biomass Supply from Alternative Cellulosic Crops and Crop Residues: A Spatial Bioeconomic Modeling Approach. (Agricultural and Applied Economics Association, 2011).

8 Aklesso, E.-M., Swinton, S., Izaurralde, R. C., Manowitz, D. H. & Zhang, X. Maintaining Environmental Quality while Expanding Energy Biomass Production: Policy Simulations from Michigan, USA. (International Association of Agricultural Economists, 2012).

9 Apezteguía, H. P., Izaurralde, R. C. & Sereno, R. Simulation study of soil organic matter dynamics as affected by land use and agricultural practices in semiarid Córdoba, Argentina. *Soil and tillage research* **102**, 101-108 (2009).

10 Beach, R. H. *et al.* Climate change impacts on US agriculture and forestry: benefits of global climate stabilization. *Environmental Research Letters* **10**, 095004 (2015).

11 Causarano, H. J. *et al.* EPIC modeling of soil organic carbon sequestration in croplands of Iowa. (2008).

12 Causarano, H. J. *et al.* Simulating field‐scale soil organic carbon dynamics using EPIC. *Soil Science Society of America Journal* **71**, 1174-1185 (2007).

13 Gelfand, I. *et al.* Sustainable bioenergy production from marginal lands in the US Midwest. *Nature* **493**, 514-517 (2013).

14 He, X., Izaurralde, R., Vanotti, M. B., Williams, J. R. & Thomson, A. M. J. J. o. e. q. Simulating long-term and residual effects of nitrogen fertilization on corn yields, soil carbon sequestration, and soil nitrogen dynamics. **35**, 1608-1619 (2006).

15 Izaurralde, R., Williams, J. R., McGill, W. B., Rosenberg, N. J. & Jakas, M. Q. Simulating soil C dynamics with EPIC: Model description and testing against long-term data. *Ecological Modelling* **192**, 362-384 (2006).

16 Izaurralde, R. *et al.* Long-term modeling of soil C erosion and sequestration at the small watershed scale. *Climatic Change* **80**, 73-90 (2007).

17 LeDuc, S. D., Zhang, X., Clark, C. M. & Izaurralde, R. C. Cellulosic feedstock production on Conservation Reserve Program land: potential yields and environmental effects. *GCB Bioenergy* (2016).

18 Schwalm, C. R. *et al.* A model‐data intercomparison of CO2 exchange across North America: Results from the North American Carbon Program site synthesis. *Journal of Geophysical Research: Biogeosciences* **115** (2010).

19 Wang, X., He, X., Williams, J., Izaurralde, R. & Atwood, J. Sensitivity and uncertainty analyses of crop yields and soil organic carbon simulated with EPIC. *Transactions of the ASABE* **48**, 1041-1054 (2005).

20 Zhang, X., Izaurralde, R. C., Arnold, J. G., Williams, J. R. & Srinivasan, R. Modifying the Soil and Water Assessment Tool to simulate cropland carbon flux: Model development and initial evaluation. *Science of the Total Environment* **463-464**, 810-822 (2013). <https://doi.org:10.1016/j.scitotenv.2013.06.056>

21 Zhang, X. *et al.* Multi-scale geospatial agroecosystem modeling: a case study on the influence of soil data resolution on carbon budget estimates. *Science of the Total Environment* **479**, 138-150 (2014).

22 West, T. O. *et al.* Cropland carbon fluxes in the United States: Increasing geospatial resolution of inventory‐based carbon accounting. *Ecological Applications* **20**, 1074-1086 (2010).

23 Srinivasan, R., Zhang, X. & Arnold, J. SWAT ungauged: hydrological budget and crop yield predictions in the Upper Mississippi River Basin. *Transactions of the ASABE* **53**, 1533-1546 (2010).

24 Wang, X., Gassman, P., Williams, J., Potter, S. & Kemanian, A. Modeling the impacts of soil management practices on runoff, sediment yield, maize productivity, and soil organic carbon using APEX. *Soil and Tillage Research* **101**, 78-88 (2008).

25 Zhang, X. *et al.* An integrative modeling framework to evaluate the productivity and sustainability of biofuel crop production systems. *Gcb Bioenergy* **2**, 258-277 (2010).

26 Zhang, X. *et al.* Regional scale cropland carbon budgets: Evaluating a geospatial agricultural modeling system using inventory data. *Environmental Modelling & Software* **63**, 199-216 (2015). <https://doi.org:10.1016/j.envsoft.2014.10.005>

27 Graham, R. L., Nelson, R., Sheehan, J., Perlack, R. & Wright, L. L. Current and potential US corn stover supplies. *Agronomy Journal* **99**, 1-11 (2007).

28 Liska, A. J. *et al.* Biofuels from crop residue can reduce soil carbon and increase CO 2 emissions. *Nature Climate Change* **4**, 398 (2014).

29 Zhang, X. *et al.* Efficient multi-objective calibration of a computationally intensive hydrologic model with parallel computing software in Python. *Environmental Modelling and Software* **46**, 208-218 (2013). <https://doi.org:10.1016/j.envsoft.2013.03.013>

30 Nichols, J. *et al.* HPC-EPIC for high resolution simulations of environmental and sustainability assessment. *Computers and Electronics in Agriculture* **79**, 112-115 (2011). <https://doi.org:10.1016/j.compag.2011.08.012>

31 Dalcin, L. D., Paz, R. R., Kler, P. A. & Cosimo, A. Parallel distributed computing using python. *Advances in Water Resources* **34**, 1124-1139 (2011).

32 Sahajpal, R., Zhang, X., Gelfand, I., Izaurralde, C. R. & Hurtt, G. Modeling representative crop rotaions in the Central United States. *Computers and Electronics in Agriculture (In Review).* (2014).

33 Farr, T. G. *et al.* The shuttle radar topography mission. *Reviews of geophysics* **45** (2007).

34 Owens, L., Bonta, J. & Shipitalo, M. USDA-ARS North Appalachian Experimental Watershed: 70-year hydrologic, soil erosion, and water quality database. *Soil Science Society of America Journal* **74**, 619-623 (2010).

35 Hao, Y. *et al.* Historic assessment of agricultural impacts on soil and soil organic carbon erosion in an Ohio watershed. *Soil Science* **166**, 116-126 (2001).

36 Karlen, D. *et al.* Field-scale watershed evaluations on deep-loess soils: I. Topography and agronomic practices. *Journal of soil and water conservation* **54**, 693-703 (1999).

37 Chung, S., Gassman, P. W., Kramer, L., Williams, J. R. & Gu, R. Validation of EPIC for two watersheds in southwest Iowa. *Journal of Environmental Quality* **28**, 971-979 (1999).

38 Kenney, I. *et al.* Soil and crop response to stover removal from rainfed and irrigated corn. *Gcb Bioenergy* **7**, 219-230 (2015).

39 Moorman, T., Cambardella, C., James, D., Karlen, D. & Kramer, L. Quantification of tillage and landscape effects on soil carbon in small Iowa watersheds. *Soil and Tillage Research* **78**, 225-236 (2004).

40 Meyer-Aurich, A., Janovicek, K., Deen, W. & Weersink, A. Impact of tillage and rotation on yield and economic performance in corn-based cropping systems. *Agronomy journal* **98**, 1204-1212 (2006).

41 Ashworth, A. J., Allen, F. L., Saxton, A. M. & Tyler, D. D. Long-term corn yield impacted by cropping rotations and bio-covers under no-tillage. *Agronomy Journal* **108**, 1495-1502 (2016).

42 Bradford, J. M. & Huang, C.-h. Interrill soil erosion as affected by tillage and residue cover. *Soil and tillage Research* **31**, 353-361 (1994).

43 Lal, R. & Moldenhauer, W. C. Effects of soil erosion on crop productivity. *Critical Reviews in Plant Sciences* **5**, 303-367 (1987).

44 Follett, R. F. & Stewart, B. A. *Soil Erosion and Crop Productivity*. (American Society of Agronomy, Inc., Crop Science Society of America, Inc., Soil Science Society of America, Inc., Publishers, 1985).

45 Den Biggelaar, C., Lal, R., Wiebe, K. & Breneman, V. Impact of soil erosion on crop yields in North America. *Advances in Agronomy* **72**, 1-52 (2001).

46 Burgess, M., Mehuys, G. & Madramootoo, C. Tillage and crop residue effects on corn production in Quebec. *Agronomy Journal* **88**, 792-797 (1996).

47 Blanco-Canqui, H. & Lal, R. Soil and crop response to harvesting corn residues for biofuel production. *Geoderma* **141**, 355-362 (2007).

48 Varvel, G. E., Vogel, K. P., Mitchell, R. B., Follett, R. & Kimble, J. Comparison of corn and switchgrass on marginal soils for bioenergy. *Biomass and bioenergy* **32**, 18-21 (2008).

49 Karlen, D. *et al.* Crop residue effects on soil quality following 10-years of no-till corn. *Soil and Tillage Research* **31**, 149-167 (1994).

50 Sims, A. L., Schepers, J. S., Olson, R. A. & Power, J. F. Irrigated corn yield and nitrogen accumulation response in a comparison of no-till and conventional till: Tillage and surface-residue variables. *Agronomy Journal* **90**, 630-637 (1998).

51 Halvorson, A. D. & Stewart, C. E. Stover removal affects no‐till irrigated corn yields, soil carbon, and nitrogen. *Agronomy Journal* **107**, 1504-1512 (2015).

52 Karlen, D. L. *et al.* Multilocation corn stover harvest effects on crop yields and nutrient removal. *BioEnergy Research* **7**, 528-539 (2014).

53 Johnson, J. M., Acosta-Martinez, V., Cambardella, C. A. & Barbour, N. W. Crop and soil responses to using corn stover as a bioenergy feedstock: Observations from the northern US Corn Belt. *Agriculture* **3**, 72-89 (2013).

54 Battaglia, M. *et al.* The broad impacts of corn stover and wheat straw removal for biofuel production on crop productivity, soil health and greenhouse gas emissions: A review. *Gcb Bioenergy* **13**, 45-57 (2021).

55 Kim, S. *et al.* Corn stover cannot simultaneously meet both the volume and GHG reduction requirements of the renewable fuel standard. *Biofuels, Bioproducts and Biorefining* **12**, 203-212 (2018).
